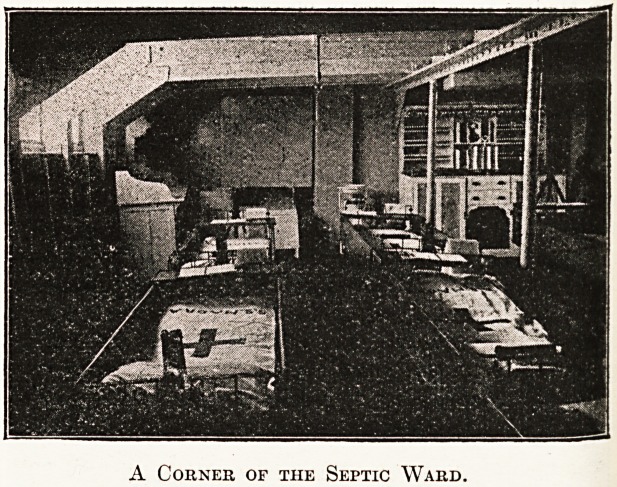# Voyages and Equipment of the Hospital Ship *Madras*

**Published:** 1915-07-24

**Authors:** 


### AN INDIAN HOSPITAL SHIP.

### Voyages and Equipment of the Hospital Ship "Madras."

FROM A CORRESPONDENT.

AFTER a lapse of six months, the hospital ship *Madras*, which left the port of Madras in November 1914, arrived in Madras from the base of operations in East Africa and the Persian Gulf, bringing in a number of sick and wounded. The vessel has done good work in serving the Indian Expeditionary Force; Colonel Giffard, C.S.I., I.M.S., has been in charge of the medical staff.

LYING AT ANCHOR ON HER RETURN TO MADRAS.

The vessel has made three trips to Mombassa and to the Persian Gulf, visiting Zanzibar, Basra, and Bombay, carrying a total of 1,200 sick and wounded. The removal of the sick and wounded into hospital has been undertaken by the Bombay Motor Ambulance and Bombay Volunteer Corps. It will be noted that the accompanying photographs provide, as it were, a general view of the vessel actually at work, from the equipment with which it starts, by way of a voyage on the high seas, to the final date of arrival and disembarkation. Many aspects of life and work on a hospital ship have been given in THE HOSPITAL, but we are glad to have this opportunity of illustrating one of the vessels which plies, not between England and France, but between two remote portions of the Empire.

At Basra the staff of the H.S. *Madras* were present on Proclamation Day parade, the G.O.C. distributing rewards to the British officers and Indian soldiers. The captured Turkish guns were also exhibited.

The Madras War Fund was established seven or eight months ago to help the fighting forces in the field so as to prevent congestion of sick and wounded at the base and to meet the economic distress which invariably results from a war. Ample provision was, of course, made in England by the establishment of the Prince of Wales' Fund and the Viceroy's Imperial Relief Fund in India. Madras subscribed her quota to the Viceroy's Fund, and in addition she has subscribed a sum of 25 lakhs for the Madras War Fund. One of the first duties of this Fund was to land in England 350 horses from Madras; the next move was the despatch of 30 motor cyclists for service in Europe in three separate batches.

THE OPERATING THEATRE, ADJOINING THE X-RAY ROOM.

There can be no question that it will be noted from this account that the equipment and accommodation provided in this vessel appear to be comprehensive, and that the enthusiasm which has accompanied its work is as notable in Madras as is any which has been manifested at home. It is, however, interesting to compare the peculiar wants which climatic conditions impose upon a vessel engaged on such a voyage as the *Madras*, between the city of that name and Mombassa, with those needed here. Realising the great distress felt in British East Africa, the Madras War Fund extended its help to the extent of £100,000. The amount was welcome for the purchase and erection of a soda-water plant, which has been a much-

WATCHING THE DISEMBARKATION.

felt want for the comfort of the troops and invalids at Mombassa. The largest item is the cost of the hire of the ship and crew, coal, and water, which runs to 1 lakh of rupees a month, with another 30,000 rupees for the maintenance of the staff and medical and surgical requisites.

The day following the arrival of the vessel the work of disembarking the sick and wounded took place, at which Lord Pentland, Governor of Madras, and his military secretary, Major Allanson, the Port Officer, the Surgeon-General, and other officers were present. Twenty men of the 2nd Loyal North Lancashires, thirty from the 63rd and 83rd Light Infantry, both Europeans and Indians, were despatched to Bangalore by a special ambulance train which had drawn up alongside the wharf. The remaining twenty were sent to the Madras General Hos-

No. 1 MAIN WARD, WITH FIFTY TO SIXTY BEDS.

pital by means of motor-cars and motor-ambulance transports. The same evening a lecture was delivered by Colonel Giffard at the Victoria Public Hall on the doings of the H.S. *Madras*, illustrated by lantern-slides, amidst a large gathering of Europeans and Indians. This meeting, at which, as we note below, Lord Pentland made an urgent appeal, affords us a characteristic example of the enthusiasm and unity which prevail throughout India on behalf of the Empire and the Allied cause. Few events, it may be hazarded, have surprised the Germans more than the cohesion and mutual loyalty of British Dependencies from the outbreak of the present struggle.

THE SEPTIC WARD. (Situated astern below the quarter deck.)

The above audience gave a fine proof of this loyalty. Lord Pentland, who presided, spoke of the necessity for continuing the work. The vessel left Madras the day following for the Persian Gulf, where she is urgently needed, carrying with her the remainder of the sick and wounded for other destinations.

### EQUIPMENT AND ACCOMMODATION.

The hospital ship *Madras* (late *Tanda*) was acquired by the Madras Government from the British India Steam Navigation Company, Limited, for the purpose of con-

A CORNER OF THE SEPTIC WARD.

veying sick and wounded Indian soldiers now at the Front back to India and to the various hospital bases. She is the gift of the public of the Madras Presidency, acquired from funds collected on account of the war. She is a new steel twin-screw steamer, built by Messrs. Stephens and Sons, of Glasgow, with a length of 430 feet, a breadth of 58 feet, and a moulded depth of 28 feet. Her engines are of the twin-screw type, and develop 5,200 horse-power, with a speed of 12 knots, while her gross tonnage amounts to 7,000 tons. She has accommodation for fifty first-class and fifty-two second-class passengers—all amidships—and is fitted throughout with fans and electric light. Into a detailed description, beyond the above, it should hardly be necessary to go, for text and illustration together in this case serve to help each other out, and, indeed, provide a picture of which the, to us in England, unusual features are readily explained by the peculiar character of the work of a hospital ship which has to carry wounded from East Africa to India. Moreover, the wounded, being Indians, have had to be provided with special accommodation. The *Madras* has large, roomy, 'tween decks and a shelter deck. She is fitted with wireless telegraphy, and possesses cold-storage accommodation. She is classed at Lloyd's 100 A1.

## Figures and Tables

**Figure f1:**
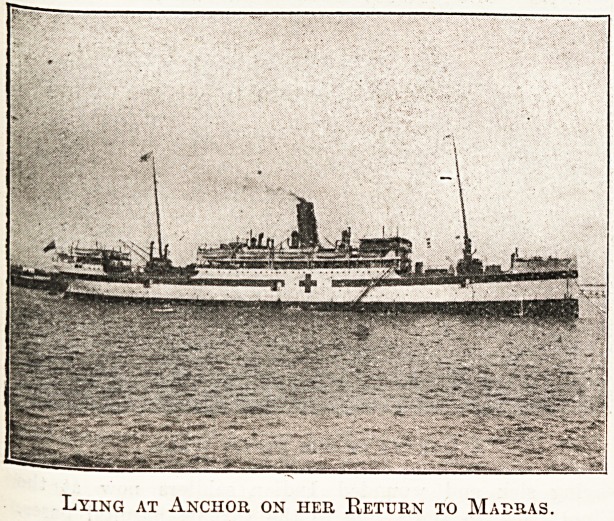


**Figure f2:**
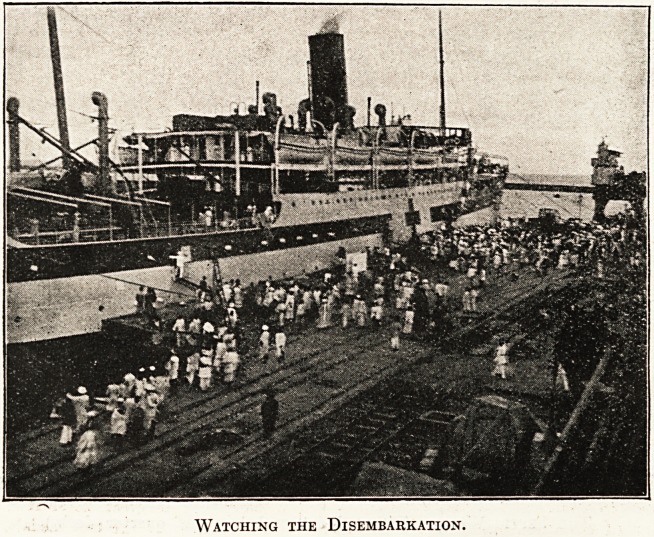


**Figure f3:**
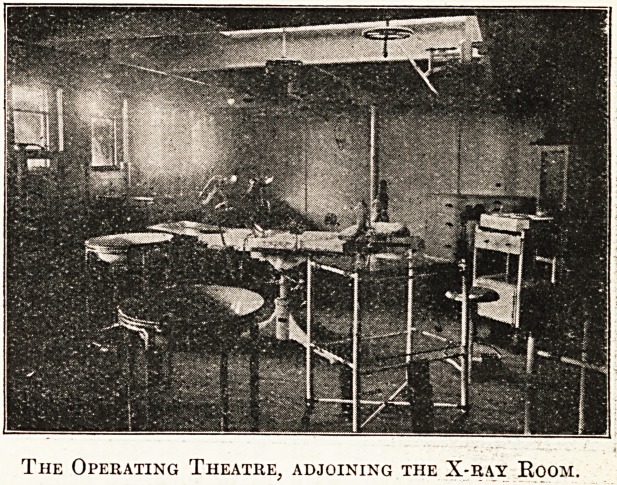


**Figure f4:**
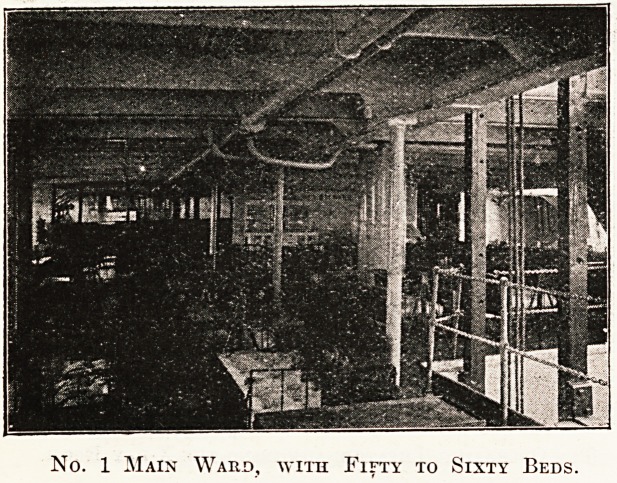


**Figure f5:**
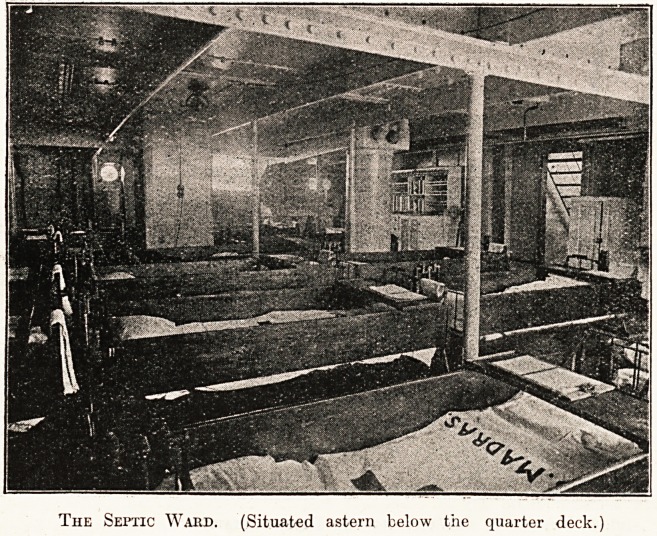


**Figure f6:**